# Genetic Structure and Demographic History Should Inform Conservation: Chinese Cobras Currently Treated as Homogenous Show Population Divergence

**DOI:** 10.1371/journal.pone.0036334

**Published:** 2012-04-27

**Authors:** Long-Hui Lin, Yan-Fu Qu, Hong Li, Kai-Ya Zhou, Xiang Ji

**Affiliations:** 1 Jiangsu Key Laboratory for Biodiversity and Biotechnology, College of Life Sciences, Nanjing Normal University, Nanjing, Jiangsu, China; 2 Hangzhou Key Lab for Animal Adaptation and Evolution, School of Life Sciences, Hangzhou Normal University, Hangzhou, Zhejiang, China; North Carolina State University, United States of America

## Abstract

An understanding of population structure and genetic diversity is crucial for wildlife conservation and for determining the integrity of wildlife populations. The vulnerable Chinese cobra (*Naja atra*) has a distribution from the mouth of the Yangtze River down to northern Vietnam and Laos, within which several large mountain ranges and water bodies may influence population structure. We combined 12 microsatellite loci and 1117 bp of the mitochondrial cytochrome *b* gene to explore genetic structure and demographic history in this species, using 269 individuals from various localities in Mainland China and Vietnam. High levels of genetic variation were identified for both mtDNA and microsatellites. mtDNA data revealed two main (Vietnam + southern China + southwestern China; eastern + southeastern China) and one minor (comprising only two individuals from the westernmost site) clades. Microsatellite data divided the eastern + southeastern China clade further into two genetic clusters, which include individuals from the eastern and southeastern regions, respectively. The Luoxiao and Nanling Mountains may be important barriers affecting the diversification of lineages. In the haplotype network of cytchrome *b*, many haplotypes were represented within a “star” cluster and this and other tests suggest recent expansion. However, microsatellite analyses did not yield strong evidence for a recent bottleneck for any population or genetic cluster. The three main clusters identified here should be considered as independent management units for conservation purposes. The release of Chinese cobras into the wild should cease unless their origin can be determined, and this will avoid problems arising from unnatural homogenization.

## Introduction

The elapid genus *Naja* comprises 28 species of non-spitting and spitting cobras and was recently split into four subgenera. Six African and Arabian non-spitting cobras inhabiting savanna and open habitats are included in the subgenus *Uraeus*, four African non-spitting cobras using forest habitats in the subgenus *Boulengerina*, seven African spitting cobras in the subgenus *Afronaja*, and 11 Asian cobras in the subgenus *Naja* [1]. Spitting adaptations appear to have evolved three times within the genus *Naja* [2]. All 11 Asian species except *N. naja* and *N. oxiana* have some degree of adaptation to spitting [1], [3]. Phylogenetic analysis of 1333 bp of mitochondrial DNA (606 for ND4 and 727 for cytochrome *b*) supports monophyly of the Asian cobras [2]. This result is consistent with that based on morphological data [4], but invalidates the hypothesis that spitting and non-spitting cobras in Asia are the outcome of two separate ancestral stocks migrating from Africa [5], [6].

**Figure 1:**
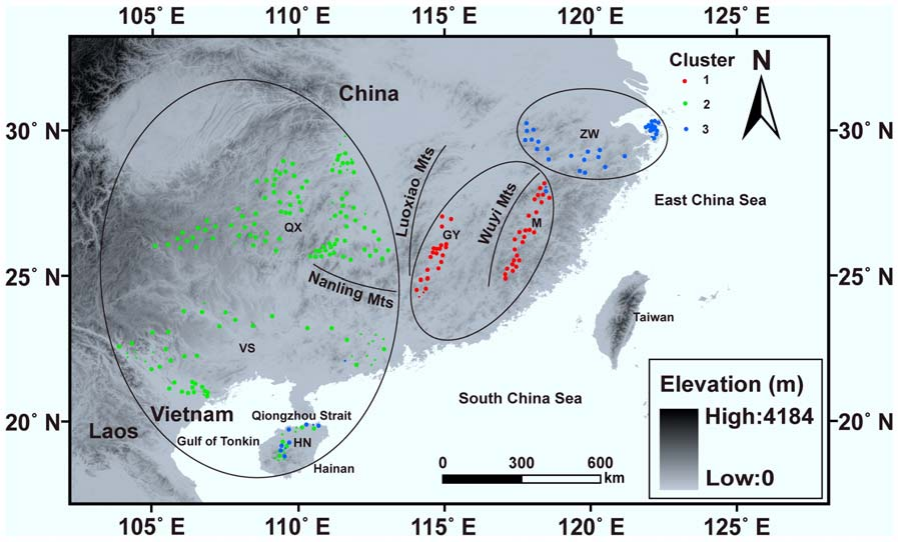
Three genetic clusters of *Naja atra* in China and Vietnam based on the admixture model in Structure. Individuals are represented by a point which is colored to reflect the cluster to which it was assigned with the highest membership coefficient. Large points represent individuals with membership coefficient *q*≥0.90 and small ones *q*<0.90. Ellipses show the three main genetic clusters. See Table 1 for sample site abbreviations.

There are two *Naja* cobras in China, the Chinese cobra (*N. atra*) and the monocled cobra (*N. kaouthia*). The Chinese cobra is found from the mouth of the Yangtze River south to northern Vietnam and Laos [7]. The cobra is of economic importance and has consequently been overexploited. It is listed as a highly vulnerable species according to the China Red Data Book of Endangered Animals [8] and is facing a realistic threat of local extinction in provinces such as Guangdong and Hainan [9]. Poachers are facing increasingly severe punishment, and great achievements have been made in artificial culture [10]. Unfortunately, however, wide populations of *N. atra* have received little conservation attention. Until now, all local populations were treated as homogenous, so that if populations were under severe threat of extinction in a part of the cobra's range, this would not necessarily ring alarm bells, as there are individuals left in other places or in farms. Within the cobra's range large mountain ranges and water bodies such as the Wuyi Mountains, Luoxiao Mountains and Nanling Mountains, Taiwan Strait and Qiongzhou Strait can be found (Fig. 1). However, it remains unknown whether these geographic barriers play a role in restricting migration and thus influencing population structure and the distribution of genetic diversity within this species.

Knowledge of population structure and genetic diversity is crucial for effective wildlife preservation because of its importance in determining the integrity and viability of wildlife populations. Moreover, these parameters can reveal more general processes acting on populations and species, such as how the population genetic structure of widespread species is formed and maintained and whether cryptic genetic groups exist within taxa [11]. The population structure and genetic diversity of a handful of snake species with large and continuous ranges has been determined for the five-paced pit-viper *Deinagkistrodon acutus* [12], the olive sea snake *Aipysurus laevis* [13], the eastern Massasauga rattlesnake *Sistrurus c. catenatus* [14], the short-tailed pit viper *Gloydius brevicaudus* [15], the Chilean long-tailed snake *Philodryas chamissonis* [16] and two hot-spring keel-back snakes, *Thermophis baileyi and T. zhaoermii* [17]. These studies generally show the existence of independent management units (MUs) for different lineages that should be taken into account for conservation plans.

Mitochondrial DNA can yield information about the historical processes behind intraspecific matrilineal relationships, and also allows insight into the importance of ongoing gene flow [18]. However, the maternally inherited haploid mitochondrial genome has a fourfold smaller effective population size than nuclear markers, which enhances the effects of genetic drift in subdivided populations and results in more rapid fixation or loss of alleles and stronger population subdivision at mitochondrial than nuclear loci [19]. While overall mutation rates tend to be higher for the mitochondrial genome [20], much of the mitochondrial genome is protein coding, potentially under selection [21] and may not always evolve sufficiently rapidly to infer levels of contemporary gene flow [22]. To reveal processes in contemporary populations influenced by both parental lineages, highly variable nuclear markers such as SNPs (single nucleotide polymorphisms) or microsatellites are required. Microsatellites are biparentally inherited and most loci appear to be selectively neutral and accumulate mutations rapidly [23].

Here, we used both mitochondrial and nuclear markers to investigate genetic processes in Chinese cobras from mainland China and Vietnam. This is the first attempt to describe genetic structure and phylogeography in this vulnerable species covering almost its entire range in mainland China and Vietnam, and is especially important because current conservation programs that release Chinese cobras reared in captivity and confiscated officially into the wild treat the population as homogenous. Several important questions regarding the population genetic characteristics of this species were addressed in this study: (1) Has genetic diversity been retained or lost following the recent and rapid decline of Chinese cobras across their range? (2) Does the cobra show genetic and population structuring across its range? (3) Are genes flowing between and within populations and are large mountain ranges and water bodies acting as geographic barriers? Answering these questions will allow us to assess the integrity, viability and population history of this taxon, thereby informing conservation efforts towards this vulnerable snake.

## Results

### Genetic variation

**Figure 2:**
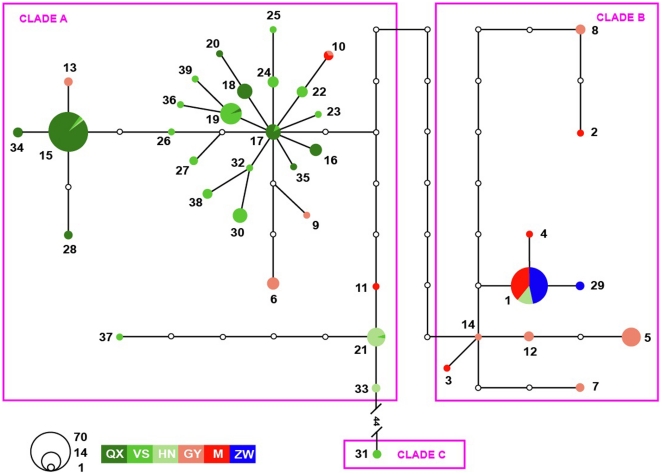
Network of 39 mitochondrial cytochrome *b* haplotypes from 269 individual *Naja atra*. The size of the circles is proportional to haplotype frequency; small open circles represent intermediate haplotypes that are not in our study. See Table 1 for sample sites abbreviations. Sites where most individuals were assigned to the same cluster on the basis of microsatellite data have gradations of the same color (s).

Cytochrome *b* gene sequences (1117 bp) from 269 individuals yielded 39 distinct haplotypes (GenBank Accession Nos EF206656, JN160643–JN160680). Eighty-six variable nucleotide sites were found, comprising 77 transitions, six transversions and three transition/transversion. While haplotypes C1 and C15 occupied a wide geographic distribution, 33 of 39 haplotypes were restricted to one population (Fig. 2). High haplotype diversity (0.863) and nucleotide diversity (0.827%) were detected for the whole population (Table 1). Individuals from ZW possessed the lowest haplotype diversity and nucleotide diversity, and individuals from M and QX the second lowest; individuals from GY, HN and VS had similar levels of mtDNA diversity (Table 1).

**Table 1:** Samples sites, sample size (*N*), number of haplotypes (*h*), haplotype diversity (Hd), nucleotide diversity (*π*), Fu's *Fs*, Tajima's *D*, sum of square deviation (SSD), Harpending's raggedness index (HRI), *τ*, number of alleles (*N*_A_), allelic richness (*AR*), number of private alleles (*N*_PA_), observed heterozygosity (*H*_O_) and expected heterozygosity (*H*_E_) for *Naja atra*.

Sample site (ID)	Mitochondrial cytochrome *b*								Microsatellite loci					
	*N*	*h*	Hd	*π* (%)	Fu's *Fs*	Tajima's *D*	SSD	HRI	*N*	*N*_A_	*AR*	*N*_PA_	*H*_O_	*H*_E_
**Eastern region**	**32**	**2**	**0.121**	**0.011**	**−0.495^NS^**	**−0.783^NS^**	**0^NS^**	**0.589^NS^**	**32**	**3.33**	**3.323**	**0**	**0.347**	**0.440**
ZW (Zhewan)	32	2	0.121	0.011	−0.495^NS^	−0.783^NS^	0^NS^	0.589^NS^	32	3.33	3.071	0	0.347	0.440
**Southeastern region**	**63**	**14**	**0.789**	**0.670**	**2.022^NS^**	**−0.318^NS^**	**0.068^NS^**	**0.138** *	**50**	**7.92**	**7.341**	**18**	**0.470**	**0.673**
M (Min)	31	6	0.353	0.332	2.688^NS^	−1.753*	0.050^NS^	0.383^NS^	28	5.92	5.340	2	0.441	0.623
GY (Ganyue)	32	9	0.780	0.800	4.318^NS^	0.574^NS^	0.079*	0.161***	22	6.92	6.541	9	0.509	0.685
**Western region**	**174**	**25**	**0.811**	**0.478**	**−2.438^NS^**	**−1.691** *	**0.043^NS^**	**0.088^NS^**	**180**	**9.83**	**7.353**	**37**	**0.514**	**0.707**
HN (Hainan)	25	4	0.617	0.732	10.280^NS^	1.963^NS^	0.214^NS^	0.365^NS^	23	5.17	4.946	3	0.496	0.638
VS (Vietnam & Southern China)	52	17	0.850	0.547	−1.324^NS^	−1.815*	0.052*	0.132**	52	8.50	6.850	7	0.631	0.753
QX (Qianxiang)	97	9	0.508	0.113	−2.005^NS^	−0.899^NS^	0.347**	0.234^NS^	105	8.00	5.502	4	0.458	0.628
**Total**	**269**	**39**	**0.863**	**0.827**	**−2.357^NS^**	**1.012^NS^**	**0.041** *	**0.050** ***	**262**	**11.42**	**8.162**	**137**	**0.485**	**0.768**

Significant values.

* 0.05≧*P*≧0.01,

** 0. 01>*P*≧0.001,

*** *P*<0.001;

NS, not significant. *AR* is calculated based on a minimum of 17 individuals. *N*_PA_ for the total sample (137) is the total number of alleles across the whole data set.

For microsatellites, 262 individuals were successfully genotyped. MICRO-CHECKER detected no evidence of null alleles or genotyping errors such as large allele dropout and stuttering. The mean number of alleles per locus (*N*_A_) was 3.33–8.50; average *H*_E_ and *H*_O_ values were 0.440–0.753 and 0.347–0.631, respectively. Individuals from ZW had the lowest *N*_A_, *H*_E_ and *H*_O_, and individuals from VS had the highest (Table 1). Hardy-Weinberg equilibrium tests showed that no population deviated from equilibrium. The test for linkage disequilibrium demonstrated that no linkage disequilibrium occurred for any pair of loci in any population (after Bonferroni correction).

### Phylogenetics and phylogeography

**Figure 3:**
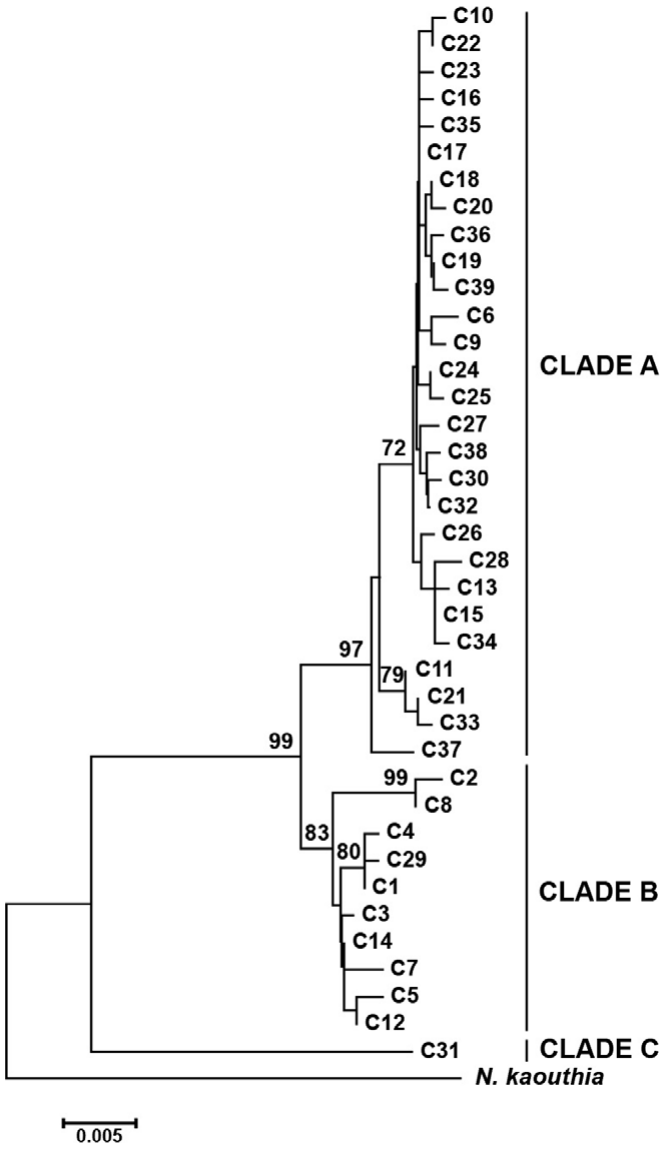
Maximum-likelihood tree for all 39 haplotypes of *Naja atra* and for one outgroup taxon. Labels are haplotype identification numbers. Values above branches indicate support for each node based on maximum likelihood. Bootstrap values below 70% are not shown.

The phylogenetic tree resulting from ML revealed three clades (Fig. 3). This finding was also supported by the median-joining network (Fig. 2). Clade A included haplotypes mainly from HN, VS and QX in the western region (in green in Fig. 2). Clade B included haplotypes mainly from M and GY in the southeastern region (in red in Fig. 2) and from the eastern region (in blue in Fig. 2). Clade C comprised only two individuals sampled from the westernmost site in the western region (Fig. 2). Clade C was divergent from the other two clades by at least 44 mutation steps (Fig. 2). Thirty-three haplotypes were unique to a single population.

Genetic distances analyses using mtDNA and microsatellite data did not produce similar results. The minimum and maximum genetic distances based on mtDNA data were from the comparisons of ZW-M (0.063) and of ZW-QX (13.836), respectively; whereas the minimum and maximum genetic distance based on microsatellite data were from the comparisons of M-GY (0.056) and of ZW-M (0.672), respectively (Table 2). The genetic distance between the ZW and M populations was its maximum based on microsatellite data but its minimum based on mtDNA cytochrome *b*.

**Table 2:** Slatkin's (1995) genetic distances (*F*_ST_/1–*F*_ST_) based on mtDNA cytochrome *b* (above diagonal) and microsatellite data (below diagonal), respectively.

	ZW	M	GY	HN	VS	QX
ZW		0.063	0.726	1.703	3.485	13.836
M	0.672		0.345	0.794	2.057	6.023
GY	0.597	0.056		0.425	0.946	2.540
HN	0.141	0.379	0.334		0.632	1.985
VS	0.202	0.303	0.247	0.068		0.448
QX	0.337	0.448	0.407	0.140	0.091	

See Table 1 for sample site abbreviations.

**Figure 4:**
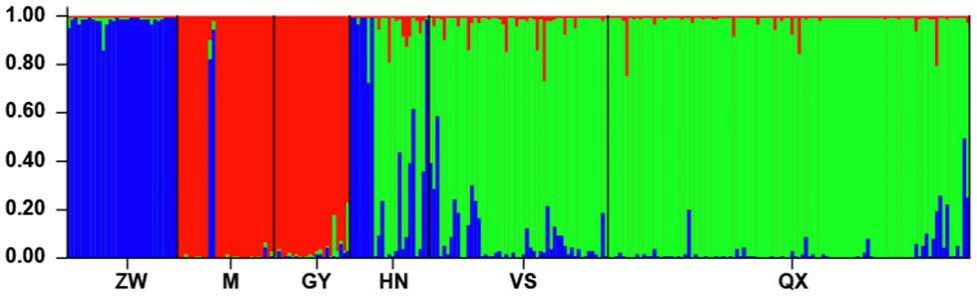
Admixture analysis of individual genotypes using Structure. Colors correspond to one of three clusters (see also Fig. 1), and each bar represents a single sample. See Table 1 for sample site abbreviations.

Cluster analyses with STRUCTURE based on microsatellite data identified two major clusters and a lower peak for *K* = 3, indicating probable substructure with three genetic groups (Fig. 4). Although the peak of Δ⊿*K* for *K* = 2 was much higher than for *K* = 3, the average likelihood values for *K* = 3 from different runs were higher than for *K* = 2 (−8901.5 and −9585.3, respectively). Therefore, we reanalyzed both clusters individually as suggested by the authors of STRUCTURE [24]. Reanalysis of cluster 1, mostly consisting of samples from M and GY, did not detect any subdivision. Repeated analysis with samples from the larger second cluster (*n* = 212, omitting snakes from M and GY) suggested the existence of two genetic subgroups (Fig. 4). Samples mainly from HN, VS and QX formed one cluster, while the other cluster consisted of samples from ZW. Each sampling site was represented in one of the three clusters with a high value of estimated membership coefficient (Table 3; Fig. 1). There was relatively few (3.8%) individuals having intermediate cluster membership (i.e. an estimated membership coefficient <0.7 in all inferred clusters), but some populations have individuals with very different cluster membership. For example, two “blue” individuals were in the red “M” sample, which could reflect very recent migrants (Fig. 1, Fig. 4). However, the varying levels of “blue” ancestry in more distant “green” samples might be a somewhat artefactual result, based on “blue” having just a subset of the diversity present in the “green” cluster. Some individuals in “green” locations might be assigned higher “blue” ancestry if they randomly happen to have some of the same alleles that drifted to high frequency in “blue”.

**Table 3:** Average estimated membership coefficients to three genetic clusters for cobras from different sampling sites in China and Vietnam.

Sample site	Average membership coefficient to cluster (*K* = 3)		
	1	2	3
ZW	0.003	0.013	**0.983**
M	**0.923**	0.009	0.069
GY	**0.961**	0.025	0.015
HN	0.031	**0.532**	0.437
VS	0.026	**0.892**	0.081
QX	0.014	**0.957**	0.029

Clusters with the highest membership coefficient are in bold, and clusters with a membership coefficient over 0.1 for given sampling sites are underlined. See Table 1 for sample site abbreviations.

### Demographic history

Although Fu's *Fs* test shows a non-significantly negative value, a significantly negative Tajima's *D* test value rejected the null hypothesis of neutral evolution and demographic equilibrium of the cytochrome *b* marker for the genetic cluster in the western region based on mtDNA data (Table 1). Moreover, many haplotypes were represented within a “star” cluster with C17 being the central haplotype in the haplotype network of cytchrome *b* (Fig. 2). This could indicate population expansion in the western region, which is supported by the mismatch distribution analysis and Rogers test [25] of sudden population expansion (Table 1). The value of *τ* for the western region was 2.36 (Table 1). Using the 95% confidence interval of 0.005–0.009 site^−1^ myr^−1^, the expansion time was estimated at 0.23–0.42 mya, falling in the interval of the late pleistocene.

BOTTLENECK tests based on microsatellite data did not provide strong evidence for a recent bottleneck for any population or genetic cluster (Table 4). The heterozygosity excess test found no significant excess under either TPM or SMM (regardless of the proportion of single-step mutations). The mode-shift test showed a normal L-shape distribution of allele frequencies in each population. The heterozygosity deficiency test found significant excess under both TPM and SMM for M, GY, QX, Cluste 1 and Cluster 2.

**Table 4:** Summary of results from BOTTLENECK for six *Naja atra* populations for the Wilcoxon's test for both the two-phase model (TPM) and stepwise mutation model (SMM) along with results from the mode-shift test.

Sample site	TPM (H deficiency/H excess)	SMM (H deficiency/H excess)	Mode-shift test
M	0.00037/1.00	0.00012/1.00	L-shape
GY	0.0031/1.00	0.0085/1.00	L-shape
HN	0.60/0.43	0.45/0.57	L-shape
VS	0.34/0.69	0.076/0.94	L-shape
QX	0.0023/1.00	0.00085/1.00	L-shape
ZW (Cluster 3)	0.77/0.26	0.54/0.48	L-shape
M-GY (Cluster 1)	0.00037/1.00	0.00012/1.00	L-shape
HN-VS-QX (Cluster 2)	0.017/0.99	0.0017/1.00	L-shape

Under the mode-shift test, an L-shaped distribution of alleles is expected in the absence of a bottleneck whereas a distribution with a shifted mode is expected in a population that has gone through a bottleneck. See Table 1 for sample site abbreviations.

## Discussion

### Genetic diversity

Our assessment of genetic variation based on mtDNA and microsatellites indicates high levels of genetic diversity in *N. atra*. For the whole population, mtDNA cytochrome haplotypic (*h* = 0.863) and nucleotide (*π* = 0.827%) diversities are higher than values reported for the Jamaican boa (*Epicrates subflavus*), a similarly vulnerable snake with fragmented distributions, using the same marker (*h* = 0.79 and *π* = 0.76%) [26]. In a study of *A. laevis* where values were not calculated for all populations sampled as a whole but for each population [13], haplotypic (*h* = 0.55–0.63) and nucleotide (*π* = 0.12–0.52%) diversities were lower for most populations of *N. atra* (Table 1). Nucleotide diversity detected in this study is lower than that (*π* = 1.41%) detected across 86 individual *D. acutus* sampled across their range in China using the entire mitochondrial ND2 gene [12].

High microsatellite diversity was also detected in *N. atra*, with a *N*_A_ of 11.42 and *H*_E_ of 0.768 (Table 1). At the species level, microsatellite diversity in *N. atra* is slightly higher than, or similar to, those reported for other snakes such as *E. subflavus* (*N*_A_ = 7.44 and *H*_E_ = 0.64) [26], *A. laevis* (*N*_A_ = 8.4 and *H*_E_ = 0.263–0.881) [13] and *S. c. catenatus* (*N*_A_ = 13.8 and *H*_E_ = 0.49–0.77) [14]. These results suggest that despite a declining population, high genetic variation remains amongst wild populations of *N. atra*, perhaps because of a large effective population size, or because bottlenecks due to very recent ecological disturbance are unlikely to dramatically reduce genetic diversity within a handful of generations unless they lead to catastrophically low population sizes.

### Genetic structure and barriers to gene flow

Our study utilized more and wider sampling locations compared to an earlier study of *N. atra* [27]. Here, phylogenetic tree and network analyses using mtDNA reveal three clades comprising individuals from the western (Clade A), eastern and southeastern (Clade B), and westernmost (Clade C) regions, and show a certain degree of geographic structure for the species. This pattern is consistent with that in snakes such as *D. acutus* distributed almost in the same region, whereby an east-west division of the whole *D. acutus* population was found [12]. With a different pattern of inheritance and rate of evolution from mtDNA, microsatellite-based analyses further divided Clade B into two genetic clusters: one included individuals from the eastern (ZW) region, and one included individuals from the southeastern (M and GY) region (Fig. 4).

Generally, the degree of genetic structure in a continuously distributed species is expected to fall somewhere between (1) a mosaic of subpopulations delimited by effective physical (e.g. mountains, rivers, roads) or ecological (e.g. prey-habitat specialization) [28] barriers to gene flow; and (2) a single genetic group with no definite boundaries or clear subdivision where gene flow between distant parts of the population may be restricted only by limited dispersal capabilities [29]. Large mountains are a dominant feature of the landscape throughout the cobra's range, and we hypothesized that these mountain ranges may affect gene flow. Based on the Bayesian clustering results the hypothesis was accepted with regard to the Luoxiao Mountains and partly accepted for the Nanling Montains, but rejected with regard to the Wuyi Mountains (Fig. 1). M and GY are located to the east of the Luoxiao Moutains and are separated from QX. As expected, significant differentiation occurred between M and QX and between GY and QX. M and GY are located to the north of the Nanling Mountains and are separated from VS. Significant differentiation occurred between M and VS, and between GY and VS. Although QX and VS are located on two different sides of the Nanling Mountains, they are only partly separated from each other by this mountain range. Consequently, significant differentiation was not found between QX and VS, possibily because of gene flow. With regard to the Wuyi Mountains, M and GY are located on two different sides of the mountain range; however, significant differentiation was not found in individuals from M and GY. More surprisingly, genetic divergences between ZW and M, and between ZW and GY are present, although no significant mountain ranges exist between these localities.

Lineage sorting between island and mainland populations has not yet been completed in the most northeastern part of the cobra's range [27], primarily because of the young age of the Zhoushan Islands which were separated from the mainland 10,000 years ago [30]. Hainan is currently separated from the mainland by the Qiongzhou Strait, which first appeared 2.5 million years ago, with the last separation occurring some 10,000 years ago [31], [32]. We found that: the haplotypes from Hainan are distributed in both Clade A and Clade B (Fig. 2, Fig. 3); haplotype C1 is shared among HN, M and ZW (Fig. 2); and haplotype C15 is shared among HN, VS and QX (Fig. 2). These findings suggest that, historically, the Qiongzhou Strait did not act as an important barrier to gene exchanges between the Hainan and mainland populations. A similar conclusion has also been drawn for the Reevese's butterfly lizard *Leiolepis reevesii* [33].

*F*_ST_ estimates based on microsatellite data also support the Bayesian clustering results (Table 2). Slatkin's genetic distances (*F*_ST_/1–*F*_ST_) between ZW and M (0.672) and between ZW and GY (0.597) are the largest when compared with values between other populations, presumably suggesting that high latitude and cool climates play a significant role in the separation of northern and southern populations of the cobra. ZW is the northern limit of the species' range, and the cobra can be very abundant in the southern region (including islands) but absent in the northern part of the region [34]. Chinese cobras never thermoregulate when air temperatures are lower than 15°C, and a prolonged exposure of cobras to temperatures lower than 9°C can be lethal [35]. The cobra may therefore have a lower capacity to tolerate cold temperatures compared to other species. Glaciation and deglaciation and the accompanying falling and rising of temperature during the Pleistocene may have greatly affected the dispersion of this species. At times of maximum glaciation most cobras in ZW moved south to seek warmer habitats, resulting in increased genetic drift, inbreeding and loss of genetic diversity. As a consequence, genetic divergence has occurred between ZW and the adjacent populations M and GY.

### Population history

There were many unobserved intermediate haplotypes missing along the long branches between the two central haplotypes (C17 and C14), whereas many haplotypes were represented within a “star” cluster in the haplotype network (Fig. 2). Is this phenomenon the outcome of a bottleneck event causing massive population extinction before subsequent recent expansion? Our answer to this question is no because BOTTLENECK tests based on microsatellites did not support a recent bottleneck for any population or genetic cluster (Table 4). Thus, the missing intermediate haplotypes could have resulted from insufficient sampling. Snakes are sensitive to changes in temperature, especially low temperatures in winter. The lack of a bottleneck and the evidence of expansion both indicate a less significant harmful effect from Quaternary glaciations on *N. atra*. In fact, no distinct Quaternary glacier landform or deposits have been found within the distribution range of *N. atra* thus far [36]. It seems likely that isolation, dispersal and expansion jointly explain the history of *N. atra*. This differs partly from conclusions drawn using the Asiatic toad *Bufo gargarizans*, an important prey item of *N. atra*. Phylogenetic analysis suggests that dispersal, instead of vicariance, dominated the history of the *B. gargarizans* species group [37]. Instead, the history of *N. atra* is somewhat similar to that of *D. acutus* where isolation, dispersal, bottlenecks and expansion jointly constitute its history [12].

### Conclusions and conservation implications

Phylogeography and population genetic studies can aid the identification of evolutionarily significant units and management units [38] and result in evidence-based conservation decision-making regarding endangered species [39]. Phylogenetic and phylogeographic analyses based on our mtDNA data support the existence of two main and a minor (comprising only two individuals) clades. Analyses based on microsatellite data further divided Clade B into two distinctive genetic clusters. Thus, besides the minor clade from the westernmost site, the three main clusters (ZW, M-GY and HN-VS-QX) should be considered as independent management units (MUs) for conservation purposes (Fig. 1). Conservation strategies such as re-introductions and translocations are required to protect or re-establish natural populations of *N. atra*, but great care should be taken to ensure that individuals are re-introduced to or translocated between appropriate populations within the same MU. Furthermore, as how much adaptive differentiation is present between these MUs or within them is unknown, we caution against long-distance transfers within a group, especially when environmental differences are apparent. In China current practices regarding the translocated release and artificial breeding of Chinese cobras in commercial farms will likely cause serious problems due to unnatural homogenization [40]. We strongly recommend that cobras should not be released into the wild unless their origin can be determined with confidence.

## Materials and Methods

### Sample collection

We employed local people to collect adult cobras larger than 900 mm snout-vent length between May and September of 2005–2010 from various localities (18°48′–30°17′N, 103°57′–122°18′E) in the eastern [Zhewan (ZW), local name for Zhejiang and Anui provinces], southeastern [Min (M), local name for Fujian province; and Ganyue (GY), local name for Jiangxi and Guangdong provinces] and western [Hainan (HN); Vietnam and southern China (VS); Qianxiang (QX), local name for Guizhou and Hunan provinces] regions of the cobra's range in mainland China and northern Vietnam (Fig. 1, Table 1). Great effort was made to avoid collecting more than one individual from the same site. The most distal 25 mm of the tail tip of each cobra was excised using a sterilized scalpel. Individual cobras were released at their site of capture after tissue sampling. Tissue samples were preserved in 95% ethanol before they were deposited at Nanjing Normal University under voucher numbers identified by locality-haplotype numbers. Our experimental procedures complied with the current laws on animal welfare and research in China, and were specifically approved by the Animal Research Ethics Committee of Nanjing Normal University (Permit No. AREC 2004-04-020). The Provincial Forestry Departments of Anhui, Fujian, Guangdong, Guangxi, Guizhou, Hainan, Hunan, Jiangxi, Yunnan and Zhejiang provided permits for capturing cobras in China. The collection of Vietnamese specimens was conducted under the ethics license from Vietnam National Museum of Nature, which was accepted by the Animal Research Ethics Committee of Nanjing Normal University.

### DNA sequencing and microsatellite genotyping

Preserved tissue samples were used to extract total genomic DNA using EasyPure Genomic DNA Extraction Kit (TransGen Biotech). DNA was resuspended in TE buffer (10 mM Tris-HCl, pH 8.0, 0.1 mM EDTA) and stored at −80°C until ready for use. The cytochrome *b* gene was amplified and sequenced according to the protocols described in Lin et al. (2008c) [27]. Twelve microsatellite loci, p8, p22, p26, p87, p88, p92, p121, p122, p124, p140, p262 and p265, were assayed following Lin et al. (2011) [41].

### MtDNA data analysis

Sequences were translated to amino acids with the program SQUINT [42] to verify if a functional mitochondrial DNA sequence was obtained and that nuclear pseudogenes were not being amplified. We compiled and aligned sequences using MEGA 5.05 [43]. We used ARLEQUIN 3.5 [44] to identify haplotypes and estimate genetic diversity within populations by haplotype (*h*) and nucleotide diversities (*π*) [45]. We tested for substitution saturation in cytochrome *b* (whole gene and each codon position separately). Within *N. atra*, signs of saturation were not present at any codon position; therefore, saturation was not considered to be a significant factor and all nucleotide positions were used in subsequent analyses.

We reconstructed a phylogenetic tree based on the maximum likelihood (ML), using *N. kaouthia* (GenBank Accession No. AF217835) as the outgroup. ML analysis was carried out by a heuristic search of 10 random addition analyses with tree-bisection-reconnection (TBR) branch swapping using PAUP 4.0 beta [46]. The GTR+I+G substitution model was selected by MODELTEST 3.7 [47] based on Akaike information criterion (AIC) [48]. The confidence level of the nodes in the ML tree was estimated using 1000 bootstrap pseudoreplicates. We also conducted a median-joining network (MJN) approach [49] to depict relationships among haplotypes. This approach has been shown to yield the best-resolved genealogies relative to other rooting and network procedures [50]. The MJN was estimated using NETWORK 4.5.0.0 [49].

We used mismatch distributions to test demographic signatures of population expansions within mtDNA lineages [51]. To compare observed distributions with those expected under the expansion model we calculated the sum of square deviation (*SSD*) and the Harpending's raggedness index [52]. Tajima's *D* test [53] and Fu's *Fs* test [54] were used to test equilibrium of the populations in ARLEQUIN 3.5. The statistics was expected to have large negative values under demographic expansion. The equation *τ* = 2*ut* [25] was used to estimate the approximate expansion time in generations (*t*), where *τ* is the date of the growth or decline measured in units of mutational time and *u* is the mutation rate per sequence and per generation. The approximate time of expansion in years was calculated by multiplying *t* by the generation time of *N. atra*. The generation time for large snakes was estimated as four years based on the approximate time at which animals mature [12], [55]. The substitution rate of mtDNA sequences had been calibrated in studies of lizards [56] and other vertebrates [57] as approximately 0.65% per million years. Based on geological events (the final emergence of the Isthmus of Panama), Wüster et al. (2002) [58] suggest a substitution rate of 0.007 site^−1^ myr^−1^ (95% confidence interval: 0.005–0.009) for cytochrome *b* within the Viperidae. We used the upper and lower values (0.005–0.009) to estimate the overall range of potential dates. Although this dating must be taken with extreme caution due to the lack of calibration of the substitution rate in *N. atra* and to the sensible overestimation of timing recent events induced by the time-dependency of molecular rates [59], it provides an approximate time frame.

### Microsatellite data analysis

All microsatellite loci were screened for null alleles and large allele dropouts using MICRO-CHECKER 2.2.3 [60]. CONVERT [61] was used to detect private alleles, which were alleles present in one population and not shared with any other. The mean number of alleles (*N*_A_) per locus and observed (*H*_O_) and expected heterozygosities (*H*_E_) were calculated using ARLEQUIN 3.5. FSTAT 2.9.3.2 [62] was used to test linkage disequilibrium and to calculate allelic richness (*AR*) on a minimum of 17 individuals. Deviations from Hardy-Weinberg equilibrium across all loci for each population were assessed using the exact probability test in GENEPOP 4.0 [63]. Significance values for multiple comparisons were adjusted using the Bonferroni correction.

Genetic distances were computed as Slatkin's (1995) genetic distance (*F*_ST_/1–*F*_ST_) derived from pairwise *F*_ST_, which were estimated for 15 comparisons between six populations [64]. Geographical distances were measured as the shortest overwater distances between pairs of locations [65]. The significance of each test was assessed using 30000 data randomizations.

A Bayesian clustering method, STRUCTURE [66], [67], was used to detect genetic clustering in the whole data set. Under STRUCTURE 2.3.3 the range of possible clusters (*K*) tested was set from 1 to 10, and 10 independent runs were carried out for each using no prior information, assumed admixture and correlated allele frequencies. The lengths of MCMC iteration and burn-in were set at 300 000 and 50 000, respectively. The true *K* is selected using the maximal value of the log likelihood [Ln Pr(*X*/*K*)] of the posterior probability of the data for a given Δ*K* [67]. Further, theΔ⊿*K* statistic, the second-order rate of change in the log probability of the data between successive values of *K*, was also estimated [68].

Demographic history based on microsatellites was assessed using two different and complementary methods. First, the Wilcoxon's sign rank test was used to examine whether populations exhibit a greater level of heterozygosity than predicted in a population at mutation-drift equilibrium. This test is most sensitive to detecting bottlenecks occurring over approximately the last 2–4 Ne generations and, for most parameters, has more power to detect more recent bottlenecks (e.g. 0.2–1Ne generations ago). Second, a mode-shift test [69] was carried out to detect a distortion of the expected L-shaped distribution of allele frequency. This test is most appropriate for detecting population declines which have occurred more recently, specifically over the last few dozen generations [69], [70]. Heterozygosity deficiency, heterozygosity excess and mode-shift tests were implemented in BOTTLENECK 1.2.02 [71]. We performed 10000 simulations in six populations and three genetic clusters under the stepwise mutation model (SMM) and the two-phase model (TPM), with 95% single step mutations and 5% multi-step mutations and a variance of 12 as recommended by Piry et al. (1999) [71]. *P*-values from the Wilcoxon's test were used as evidence for bottlenecks and were assessed for significance at the 0.05 level.

## References

[1] Wallach V, Wüster W, Broadley DG (2009). In praise of subgenera: taxonomic status of cobras of the genus *Naja* Laurenti (Serpentes: Elapidae).. Zootaxa.

[2] Wüster W, Crookes S, Ineich I, Mané Y, Pook CE (2007). The phylogeny of cobras inferred from mitochondrial DNA sequences: evolution of venom spitting and the phylogeography of the African spitting cobras (Serpentes: Elapidae: *Naja nigricollis* complex).. Mol Phylogenet Evol.

[3] Wüster W, Thorpe RS (1992). Asiatic cobras: population systematics of the *Naja naja* species complex (Serpentes: Elapidae) in India and Central Asia.. Herpetologica.

[4] Szyndlar Z, Rage JC (1990). West Palearctic cobras of the genus *Naja* (Serpentes: Elapidae): interrelationships among extinct and extant species.. Amphibia-Reptilia.

[5] Ineich I (1995). Etat actuel de nos connaissances sur la classification des serpents venimeux.. Bull Soc Herpétol France 1995.

[6] Minton SA, Harris JB (1986). Origins of poisonous snakes: evidence from plasma and venom proteins.. Natural toxins: animal, plant and microbial.

[7] Wüster W, Golay P, Warrell DA (1997). Synopsis of recent developments in venomous snake systematics.. Toxicon.

[8] Zhao EM, Zhao EM (1998). *Naja atra* Cantor.. China red data book of endangered animals (Amphibia and Reptilia).

[9] Lin LH, Li H, An H, Ji X (2008). Do temperature fluctuations during incubation always play an important role in shaping the phenotype of hatchling reptiles?. J Therm Biol.

[10] Tan QY, Guo TG, Gong XG, Gong XJ, Guo DZ (2009). Artificial domestication and breeding technology of *Naja atra*.. J Snake.

[11] Tammeleht E, Remm J, Korsten M, Davison J, Tumanov I (2010). Genetic structure in large, continuous mammal populations: the example of brown bears in northwestern Eurasia.. Mol Ecol.

[12] Huang S, He SP, Peng ZG, Zhao K, Zhao EM (2007). Molecular phylogeography of endangered sharp-snouted pitviper (*Deinagkistrodon acutus*; Reptilia, Viperidae) in mainland China.. Mol Phylogenet Evol.

[13] Lukoschek V, Waycott M, Keogh JS (2008). Relative information content of polymorphic microsatellites and mitochondrial DNA for inferring dispersal and population genetic structure in the olive sea snake, *Aipysurus laevis*.. Mol Ecol.

[14] Chiucchi JE, Gibbs HL (2010). Similarity of contemporary and historical gene flow among highly fragmented populations of an endangered rattlesnake.. Mol Ecol.

[15] Ding L, Gan XN, He SP, Zhao EM (2011). A phylogeographic, demographic and historical analysis of the short-tailed pit viper (*Gloydius brevicaudus*): evidence for early divergence and late expansion during the Pleistocene.. Mol Ecol.

[16] Sallaberry-Pincheira N, Garin CF, González-Acuña D, Sallaberry MA, Vianna JA (2011). Genetic divergence of Chilean long-tailed snake (*Philodryas chamissonis*) across latitudes: conservation threats for different lineages.. Divers Distrib.

[17] Huang S, Liu SY, Guo P, Zhang YP, Zhao EM (2009). What are the closest relatives of the hot-spring snakes (Colubridae, *Thermophis*), the relict species endemic to the Tibetan Plateau?. Mol Phylogenet Evol.

[18] Avise JC (2000). Phylogeography: the history and formation of species.

[19] Birky CW, Maruyama T, Fuerst PA (1983). An approach to population and evolutionary genetic theory for genes in mitochondria and chloroplasts, and some results.. Genetics.

[20] Brown WM, George MJ, Wilson AC (1979). Rapid evolution of animal mitochondrial DNA.. Proc Natl Acad Sci USA.

[21] Ballard JWO, Kreitman M (1995). Is mitochondrial DNA a strictly neutral marker?. Trends Ecol Evol.

[22] Angers B, Bernatchez L (1998). Combined use of SMM and non-SMM methods to infer fine structure and evolutionary history of closely related brook charr (*Salvelinus fontinalis*, Salmonidae) populations from microsatellites.. Mol Biol Evol.

[23] Balloux F, Lugon-Moulin N (2002). The estimation of population differentiation with microsatellite markers.. Mol Ecol.

[24] Pritchard JK, Wen W (2004). Documentation for structure software: Version 2.. http://pritch.bsd.uchicago.edu/software/readme_structure2.pdf.

[25] Rogers AR, Harpending H (1992). Population growth makes waves in the distribution of pairwise genetic differences.. Mol Biol Evol.

[26] Tzika AC, Remy C, Gibson R, Milinkovitch MC (2009). Molecular genetic analysis of a captive-breeding program: the vulnerable endemic Jamaican yellow boa.. Conser Genet.

[27] Lin LH, Zhao Q, Ji X (2008). Conservation genetics of the Chinese cobra (*Naja atra*) using mitochondrial DNA sequences.. Zool Sci.

[28] Musiani M, Leonard JA, Cluff HD, Gates CC, Mariani S (2007). Differentiation of tundra/taiga and boreal coniferous forest wolves: genetics, coat colour and association with migratory caribou.. Mol Ecol.

[29] Epperson BK (2003). Geographical genetics: monographs in population biology, 38.

[30] Wang JT, Wang PS (1980). Relationship between sea-level changes and climatic fluctuations in east China since late Pleistocene.. Acta Geogr Sin.

[31] Chen XD, Fan SQ (1988). Late quaternary deposition and environment in the sea area of Northwest Hainan Island.. Tropical Ocean.

[32] Zhao HT, Zhang QM, Song CJ (1999). Geomorphology and environment of the south China coast and the south China sea islands.

[33] Lin LH, Ji X, Diong CH, Du Y, Lin CX (2010). Phylogeography and population structure of the Reevese's butterfly lizard (*Leiolepis reevesii*) inferred from mitochondrial DNA sequences.. Mol Phylogenet Evol.

[34] Ji X, Wang ZW (2005). Geographic variation in reproductive traits and trade-offs between size and number of eggs of the Chinese cobra (*Naja atra*).. Biol J Linn Soc.

[35] Ji X, Chen HL, Du WG, Zhu BQ (2002). Radiotelemetry of thermoregulation and thermal tolerance on Chinese cobras (*Naja atra*) overwintering in a laboratory enclosure.. Acta Zool Sin.

[36] Li JJ, Shu Q, Zhou SZ, Zhao ZJ, Zhang JM (2004). Review and prospects of quaternary glaciation research in China.. J Glaciol Geocryol.

[37] Fu JZ, Weadick CJ, Zeng XM, Wang YZ, Liu ZJ (2005). Phylogeographic analysis of the *Bufo gargarizans* species complex: a revisit.. Mol Phylogenet Evol.

[38] Moritz C (1994). Defining ‘Evolutionarily Significant Units’ for conservation.. Trends Ecol Evol.

[39] Szaro RC (2008). Endangered species and nature conservation: science issues and challenges.. Integr Zool.

[40] Lin HC, Li SH, Fong J, Lin SM (2008). Ventral coloration differentiation and mitochondrial sequences of the Chinese cobra (*Naja atra*) in Taiwan.. Conser Genet.

[41] Lin LH, Mao LX, Luo X, Qu YF, Ji X (2011). Isolation and characterization of microsatellite loci in the Chinese cobra *Naja atra* (Elapidae).. Int J Mol Sci.

[42] Goode M, Rodrigo AG (2007). SQUINT: A multiple alignment program and editor.. Bioinformatics.

[43] Tamura K, Peterson D, Peterson N, Stecher G, Nei M (2011). MEGA5: molecular evolutionary genetics analysis using maximum likelihood, evolutionary distance, and maximum parsimony methods.. Mol Biol Evol.

[44] Excoffier L, Lischer HEL (2010). Arlequin suite ver 3.5: a new series of programs to perform population genetics analyses under Linux and Windows.. Mol Ecol Resour.

[45] Nei M (1987). Molecular evolutionary genetics.

[46] Swofford DL (2003). PAUP* phylogenetic analysis using parsimony (*and other methods), Version 4.

[47] Posada D, Crandall K (1998). Modeltest: testing the model of DNA substitution.. Bioinformatics.

[48] Akaike H (1974). A new look at the statistical model identification.. IEEE T Automat Contr.

[49] Bandelt HJ, Forster P, Rohl A (1999). Median-joining networks for inferring intra-specific phylogenies.. Mol Biol Evol.

[50] Cassens I, Van Waerebeek K, Best PB, Crespo EA, Reyes J (2003). The phylogeography of dusky dolphins (*Lagenorhynchus obscurus*): a critical examination of network methods and rooting procedure.. Mol Ecol.

[51] Rogers AR (1995). Genetic evidence for a Pleistocene population explosion.. Evolution.

[52] Harpending HC (1994). Signature of ancient population growth in a low-resolution mitochondrial DNA mismatch distribution.. Hum Biol.

[53] Tajima F (1989). Statistical method for testing the neutral mutation hypothesis by DNA polymorphism.. Genetics.

[54] Fu YX (1997). Statistical tests of neutrality of mutations against population growth, hitchhiking and background selection.. Genetics.

[55] Huang S, Huang JT (2003). Artificial propagation of the five-paced pitviper (*Deinagkistrodon acutus*).. Acta Zool Sin.

[56] Macey JR, Schulte JA, Ananjeva NB, Larson A, Rastegar-Pouyani N (1998). Phylogenetic relationships among agamid lizards of the *Laudakia caucasia* complex: testing hypotheses of fragmentation and an area cladogram for the Iranian Plateau.. Mol Phylogenet Evol.

[57] Macey JR, Schulte JA, Larson A, Fang ZL, Wang YZ (1998). Phylogenetic relationships of toads of the *Bufo bufo* complex from the eastern escarpment of the Tibetan Plateau: a case of vicariance and dispersal.. Mol Phylogenet Evol.

[58] Wüster W, Salomão MDG, Quijada-Mascareñas JA, Thorpe RS, BBBSP, Schuett GW, Höggren MH, Douglas ME, Greene HW (2002). Origins and evolution of the South American pitviper fauna: evidence from mitochondrial DNA sequence analysis.. Biology of vipers.

[59] Ho SYW, Larson G (2006). Molecular clocks: when times are a-changin'.. Trends Genet.

[60] Van Oosterhout C, Hutchinson WF, Wills DPM, Shipley P (2004). MICRO-CHECKER: software for identifying and correcting genotyping errors in microsatellite data.. Mol Ecol Notes.

[61] Glaubitz JC (2004). CONVERT: a user friendly program to reformat diploid genotypic data for commonly used population genetic software packages.. Mol Ecol Notes.

[62] Goudet J (2001). FSTAT, a program to estimate and test gene diversities and fixation indices (version 2.9.3).. http://www.unil.ch/izea/softwares/fstat.html.

[63] Rousset F (2008). GENEPOP'007: a complete re-implementation of the GENEPOP software for Windows and Linux.. Mol Ecol Resour.

[64] Slatkin M (1995). A measure of population subdivision based on microsatellite allele frequencies.. Genetics.

[65] Slatkin M (1993). Isolation by distance in equilibrium and non-equilibrium populations.. Evolution.

[66] Falush D, Stephens M, Pritchard JK (2003). Inference of population structure using multilocus genotype data: linked loci and correlated allele frequencies.. Genetics.

[67] Pritchard JK, Stephens M, Donnelly P (2000). Inference of population structure using multilocus genotype data.. Genetics.

[68] Evanno G, Regnaut S, Goudet J (2005). Detecting the number of clusters of individuals using the software STRUCTURE: a simulation study.. Mol Ecol.

[69] Luikart G, Allendorf FW, Cornuet JM, Sherwin WB (1998). Distortion of allele frequency distributions provides a test for recent population bottlenecks.. J Hered.

[70] Cornuet JM, Luikart G (1996). Description and power analysis of two tests for detecting recent population bottlenecks from allele frequency data.. Genetics.

[71] Piry S, Luikart G, Cornuet JM (1999). Bottleneck: a computer program for detecting recent reductions in the effective population size using allele frequency data.. J Hered.

